# Maternal fructose drives placental uric acid production leading to adverse fetal outcomes

**DOI:** 10.1038/srep25091

**Published:** 2016-04-29

**Authors:** Zeenat A. Asghar, Alysha Thompson, Maggie Chi, Andrew Cusumano, Suzanne Scheaffer, Noor Al-Hammadi, Jessica L. Saben, Kelle H. Moley

**Affiliations:** 1 Department of Obstetrics & Gynecology, Washington University in St. Louis School of Medicine, St. Louis, Missouri, USA; 2 Department of Biostatistics, Washington University in St. Louis School of Medicine, St. Louis, Missouri, USA

## Abstract

Maternal metabolic diseases increase offspring risk for low birth weight and cardiometabolic diseases in adulthood. Excess fructose consumption may confer metabolic risks for both women and their offspring. However, the direct consequences of fructose intake *per se* are unknown. We assessed the impact of a maternal high-fructose diet on the fetal-placental unit in mice in the absence of metabolic syndrome and determined the association between maternal serum fructose and placental uric acid levels in humans. In mice, maternal fructose consumption led to placental inefficiency, fetal growth restriction, elevated fetal serum glucose and triglyceride levels. In the placenta, fructose induced *de novo* uric acid synthesis by activating the activities of the enzymes AMP deaminase and xanthine oxidase. Moreover, the placentas had increased lipids and altered expression of genes that control oxidative stress. Treatment of mothers with the xanthine oxidase inhibitor allopurinol reduced placental uric acid levels, prevented placental inefficiency, and improved fetal weights and serum triglycerides. Finally, in 18 women delivering at term, maternal serum fructose levels significantly correlated with placental uric acid levels. These findings suggest that in mice, excess maternal fructose consumption impairs placental function via a xanthine oxidase/uric acid-dependent mechanism, and similar effects may occur in humans.

Consumption of sugar and high-fructose corn syrup is on the rise and has been implicated in development of metabolic syndrome, which includes obesity, dyslipidemia, hypertension, and diabetes^1^,^2^. Moreover, in both rats and humans, excess fructose consumption leads to hyperuricemia and insulin resistance^3^,^4^, which are strongly associated with metabolic syndrome and type 2 diabetes.

Fructose consumption may contribute to metabolic disease because of the way it is metabolized. Unlike glucose, whose metabolism is tightly regulated to produce ATP, the bulk of ingested fructose is extracted at first pass in the liver, where it is rapidly converted to fructose-1-phosphate by phosphofructokinase^5^. Conversion of fructose to fructose-1-phosphate leads to cellular depletion of ATP and activation of AMP deaminase (AMPD); AMPD converts AMP to xanthine, which is then converted to uric acid by xanthine oxidase^1^. This has two consequences. First, whereas extracellular uric acid can act as a potent antioxidant and is beneficial, excess intracellular uric acid can lead to oxidative stress and cellular dysfunction^1^,^6^,^7^. Second, excess uric acid can lead to increased *de novo* lipogenesis, thereby causing lipotoxicity and promoting oxidative stress and inflammation^7^,^8^. This effect may explain why excess fructose intake can promote accumulation of intrahepatic triglyceride in both healthy and type 2 diabetic subjects and lead to the development or worsening of non-alcoholic fatty liver disease^9^,^10^,^11^.

Maternal metabolic diseases are associated with increased obstetric complications such as preeclampsia, gestational diabetes, poor placentation, and intrauterine growth restriction. However, in the absence of metabolic diseases, consumption of sucrose and sugar-sweetened beverages can also increase the risk for developing preeclampsia^12^,^13^. It is well established that nutritional and other environmental exposures during fetal development can permanently affect the composition, homeostatic systems, and functions of multiple organs and systems^14^,^15^. Thus, pregnancy complications predispose the offspring to poor metabolic health in adulthood^16^. Accordingly, many human and rodent studies have shown that changes to maternal diet can lead to an adverse intrauterine environment that impairs fetal development and increases offspring risk of future diseases (reviewed in)^17^.

We have little understanding of how fructose intake during pregnancy affects placental function and fetal development. Whereas fructose intake is associated with components of metabolic syndrome^18^, many of these alterations were observed in experiments in which humans consumed excess fructose under hyper-caloric conditions^19^. However, the deleterious effects of fructose do occur in the absence of excess energy intake in mice^20^. Studies examining the effects of fructose consumption in rats during the pre- and early post-natal periods showed maternal metabolic derangements with either some^21^,^22^,^23^ or no metabolic consequence in the offspring^24^.

We recently showed that in C57BL6 mice, exposure to a high-fructose diet conferred few of the phenotypes associated with metabolic syndrome^25^ but did lead to smaller litter sizes, which was, at least in part, due to a defect in decidualization. This model thus allows us to investigate the effects of a high-fructose diet on reproductive outcomes without confounding by the full metabolic syndrome. Here, we demonstrate that HFrD-exposed mice have placental defects and investigate the mechanism by which this occurs. Additionally, to assess human relevance of our mouse data, we examined the association between maternal serum fructose and placental uric acid in a small cohort of women delivering at term by cesarean.

## Results

### Metabolic effects of high-fructose diet on mice during pregnancy

We previously demonstrated that consumption of a high-fructose diet (HFrD) led to impaired glucose tolerance but not weight gain, insulin resistance, or triglyceridemia in pseudo-pregnant C57BL6 female mice^25^. Here, we asked what effect HFrD consumption had on virgin females. As anticipated, HFrD-fed mice consumed food at a similar rate as control chow-fed mice and exhibited impaired glucose tolerance but not weight gain, insulin resistance, or triglyceridemia at the end of the six-week feeding paradigm (Supplementary Fig. S1). Thus, HFrD feeding in mice led to only a few features of metabolic syndrome with no gross dietary deficiencies, particularly protein. This enabled us to examine the direct effects of elevated fructose on pregnancy.

We next mated the mice to males maintained on a control chow diet and measured maternal and fetal serum values of metabolites on day post-coital (dpc) 18.5 (Table 1). Serum glucose and fructose levels were significantly elevated in HFrD-fed mothers and in their fetuses. Serum triglyceride levels were decreased in the HFrD-fed mothers but increased in their fetuses. This finding led us to investigate the mothers’ physiological adaptations to pregnancy. In control chow-fed mice, serum triglyceride (0.54 ± 0.03 mM vs. 0.32 ± 0.02 mM) and cholesterol (1.39 ± 0.1 mM vs. 0.79 ± 0.07 mM) levels were higher in pregnant than non-pregnant mice as expected as these are normal physiological responses during pregnancy^26^. In HFrD-fed mice, levels of serum triglycerides were also higher in pregnant than in non-pregnant mice (0.38 ± 0.03 mM vs. 0.30 ± 0.01 mM), but the difference was significantly smaller than in controls. Additionally, cholesterol levels (1.01 ± 0.03 mM vs. 0.92 ± 0.07 mM) did not differ in pregnant and non-pregnant HFrD-fed mice. This observation suggests that some physiological adaptations to pregnancy were perturbed in the HFrD-fed mice. Other metabolic features such as insulin levels, insulin sensitivity (QUICKI), uric acid levels (Table 1) and maternal blood pressure (Supplementary Table S1) were equivalent between HFrD- and control chow-fed pregnant mice. However, HFrD- but not control chow-fed pregnant mice had hepatic steatosis at dpc 18.5 (Supplementary Fig. S2).

### Maternal high-fructose diet leads to fetal growth restriction and placental inefficiency

**Figure 1:**
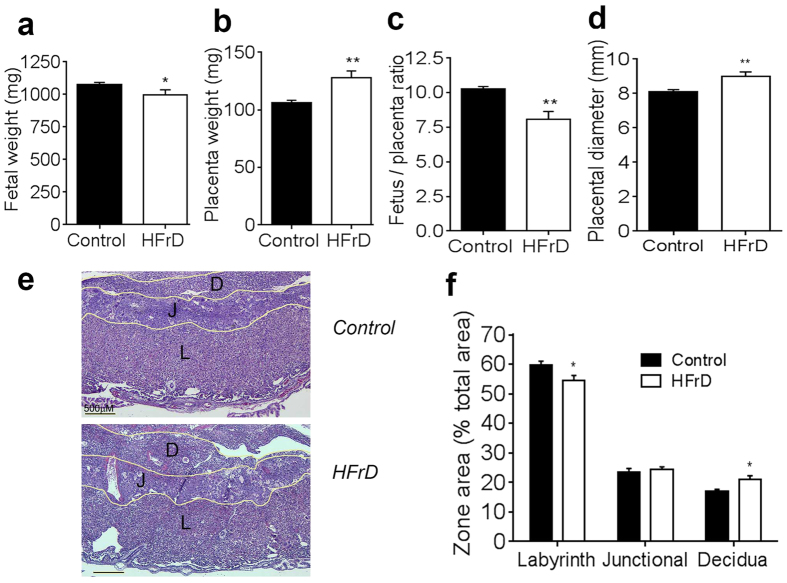
Maternal high-fructose diet decreases placental efficiency. **(a)** Fetal weights, **(b)** placental weights, **(c)** fetal to placental weight ratios, **(d)** placental diameter, **(e)** representative H&E staining of placental sections at dpc 18.5, and **(f)** quantification of placenta zone areas. D, decidua; J, Junctional zone; L, Labyrinth; n = 7–8 mothers in each group. Data are presented as mean and standard error of the mean. *P < 0.05, **P < 0.01 by Student’s *t*-test.

We found that fetuses of mothers that consumed HFrD had smaller weights than fetuses of control chow-fed mothers (Fig. 1a), but crown-rump lengths were similar between the two groups (*data not shown*). Placental weights were significantly larger on average in HFrD-fed mice than in chow-fed control mice (Fig. 1b). This resulted in a significant decrease in the fetal-to-placental weight ratio in HFrD mice (Fig. 1c), suggesting that HFrD consumption led to placental inefficiency. Moreover, placentas of HFrD-fed mice had a larger diameter than those of control chow-fed mice (Fig. 1d) and showed zone-specific changes in area (Fig. 1e). The maternally derived decidua was larger in HFrD mice, whereas the labyrinth zone, which is primarily responsible for maternal-fetal exchange, was smaller (Fig. 1f), consistent with the presence of placental inefficiency.

### Fructose drives uric acid production in the placenta

**Figure 2:**
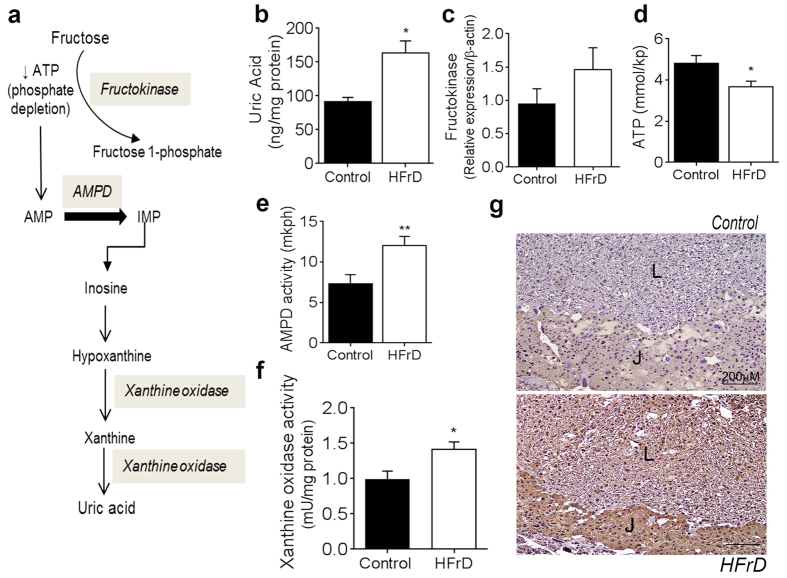
Maternal high-fructose diet increases placental uric acid levels. **(a)** Fructose metabolism drives uric acid production; schematic adapted from^1^. **(b–f)** Intra-placental levels of (**b**) uric acid, **(c)** fructokinase mRNA determined by quantitative PCR, **(d)** ATP levels, **(e)** AMPD activity, and **(f)** xanthine-oxidase activity. n = 8–10 mothers in each group with 3–4 placental samples pooled for each mouse. **(g)** Representative images of immunohistochemical staining of placental sections for xanthine oxidase; L = labyrinth zone and J = junctional zone. AMPD, adenosine monophosphate deaminase; IMP, inosine monophosphate. Data are presented as mean and standard error of the mean. *P < 0.05, **P < 0.01 by Student’s *t-*test.

Given that fructose metabolism drives uric acid production in the liver (Fig. 2a)^1^, we wondered whether a similar effect occurred in the placentas of HFrD-fed mice. Indeed, placental uric acid was significantly higher in placentas of HFrD-fed mice than in those of control chow-fed mice (Fig. 2b). Fructokinase was expressed in the mouse placenta, but it did not increase significantly in HFrD-fed mice (Fig. 2c). Consistent with the fructose-uric acid pathway (Fig. 2a), placentas of HFrD-fed mice had lower levels of ATP (Fig. 2d) and higher AMPD activity (Fig. 2e) than those of control chow-fed mice. AMPD converts AMP to inosine monophosphate, which leads to the production of xanthine via xanthine oxidase (Fig. 2a). We found that xanthine oxidase activity (Fig. 2f) and expression (Fig. 2g) were higher in placentas from HFrD-fed mothers than in placentas from control chow-fed mothers. In rodents, the hepatic enzyme uricase further converts uric acid to a harmless by-product, allantoin^27^. We found that uricase was expressed in the liver but not in the placenta (Supplementary Fig. S3). Together with our observation that HFrD-fed mothers did not have elevated serum uric acid (Table 1), these data indicate that excess fructose consumption induced *de novo* placental production of uric acid through increased AMPD and xanthine oxidase activity. In addition, due to the lack of uricase expression in the placenta, uric acid accumulation occurs.

### Maternal high-fructose diet causes lipid accumulation and oxidative stress in the placenta

**Figure 3:**
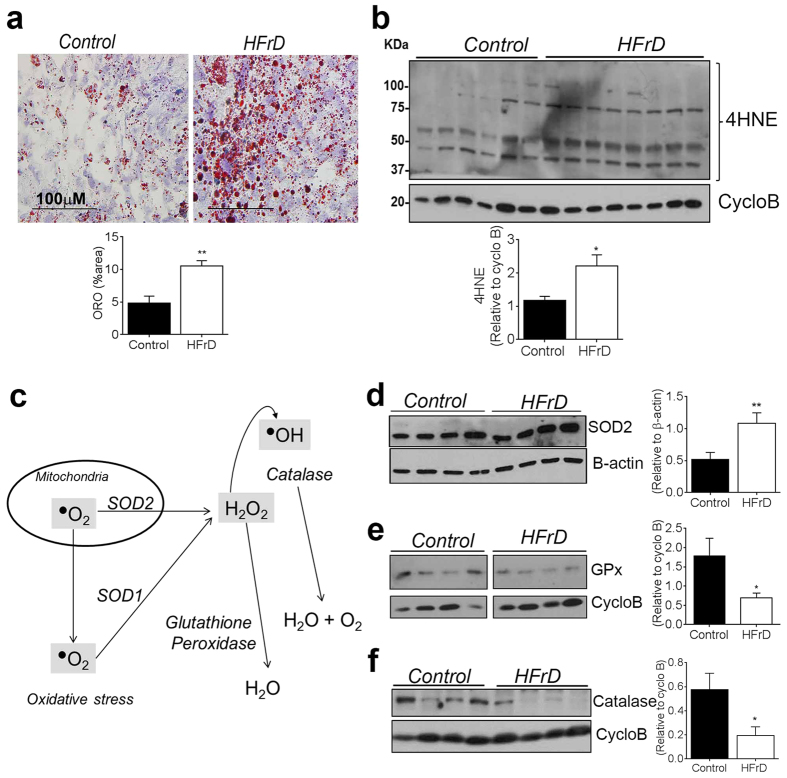
Maternal high-fructose diet leads to lipid accumulation and oxidative stress in the placenta. **(a)** Representative image of Oil-red-O stained sections of placental decidua from control and HFrD-fed mice on dpc 18.5 (top) and quantification by imageJ software (bottom). n = 5 mothers in each group, **(b)** Representative immunoblot (top) and quantification (bottom) of 4-HNE-modified proteins in placentas from control and HFrD-fed mice, **(c)** Schematic depicting role of enzymes that contribute to oxidative damage, modified from^53^. (**d–f**) Representative western blots (left) and quantitation of expression (right) of (**d**) superoxide dismutase 2 (SOD2) relative to beta-actin, **(e)** Glutathione peroxidase, GPx (samples were run on the same gel but were non-contiguous) relative to cyclophilin B, and **(f)** Catalase, relative to cyclophilin B. Each lane contains pooled placental samples from one mother; n = 6–8 mothers per group. Data are presented as mean and standard error of the mean. *P < 0.05, ** *P* < 0.01 by Student’s *t*-test.

Fructose can induce lipid accumulation in hepatocytes in a uric acid-dependent manner^8^, and we found that placentas of HFrD-fed mice had significantly more triglycerides, particularly in the decidua, than those of control-chow-fed mice (Fig. 3a). Accumulation of excess triglycerides in cells leads to oxidative stress^28^ and oxidative stress is often observed in placentas from growth-restricted fetuses^29^. Therefore, we hypothesized that uric acid-induced oxidative stress contributes to the smaller fetal size in HFrD-exposed offspring. To explore this possibility, we examined levels of protein modification by 4-Hydroxynonenal (4-HNE), a diffusible product of lipid peroxidation that has been implicated as a key mediator of oxidative stress-induced cell death^30^. We found that placentas of HFrD-fed mice had a significant increase in levels of 4-HNE-modified proteins (Fig. 3b), suggesting that excess fructose induces a pro-oxidant environment within the placenta. To further characterize the oxidative state of fructose-exposed placentas, we measured levels of the antioxidant enzymes superoxide dismutase 1 and 2 (SOD1 and SOD2), which convert superoxide radicals to hydrogen peroxide and oxygen, and glutathione peroxidase and catalase, which detoxify excess hydrogen peroxide (Fig. 3c). Levels of both SOD2 (Fig. 3d) and SOD1 (*data not shown*) were higher in placentas of HFrD-fed mice than in those of control chow-fed mice, but only the SOD2 difference was statistically significant. Levels of both glutathione peroxidase (Fig. 3e) and catalase (Fig. 3f) were significantly lower in placentas of HFrD-fed mice than in controls. This deficiency in glutathione peroxidase and catalase coupled with increased SOD expression suggests that optimal protection from oxidative stress may be reduced and inefficient in HFrD-exposed placentas.

### Allopurinol rescues adverse placental and fetal outcomes

**Figure 4:**
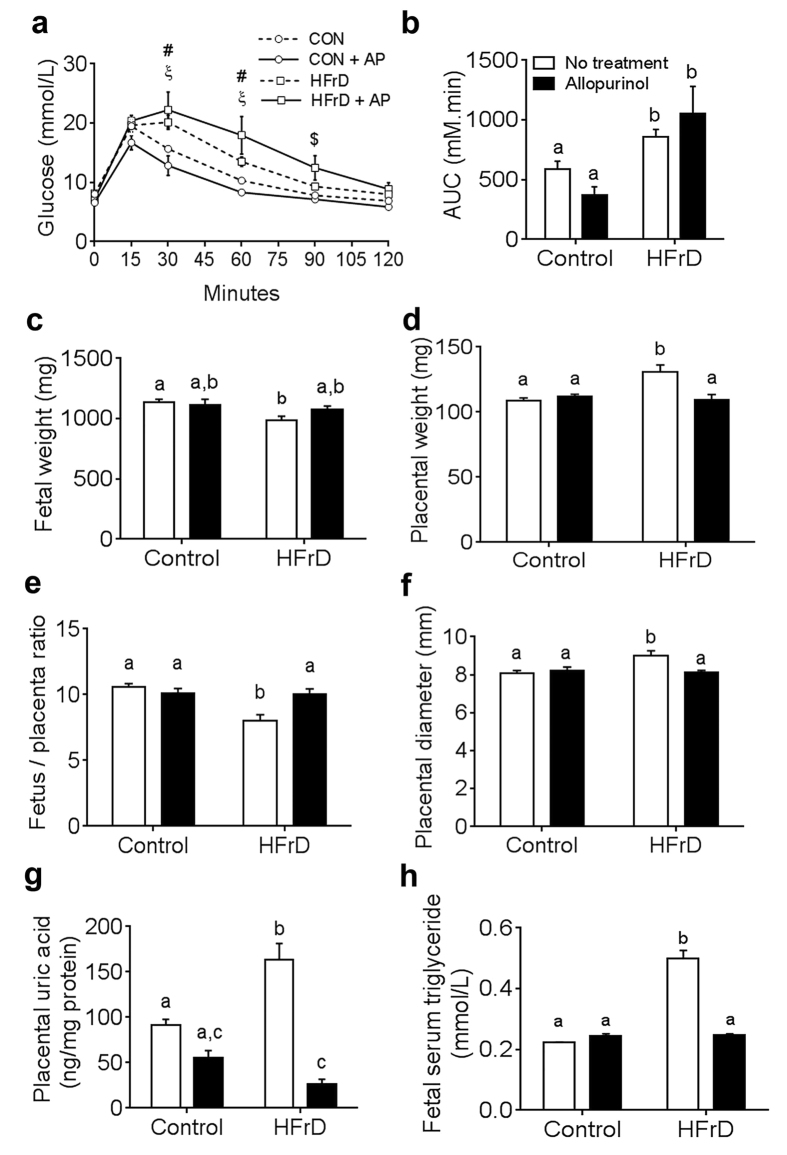
Allopurinol prevents adverse fetal-placental phenotypes. Mice were untreated (white bars) or treated with allopurinol (+AP, black bars). **(a)** Glucose tolerance test after 3 weeks of allopurinol treatment; n = 5 in allopurinol group, n = 12 in untreated group, ^#^*P* < 0.001 control vs. HFrD, ^ξ^*P* < 0.0001 control vs. HFrD+AP, ^$^*P* < 0.01 control vs. HFrD+AP. **(b)** Quantification of area under the curve (AUC) from data in (**a**). (**c–h**) Measurements on dpc 18.5 of **(c)** fetal weight, **(d)** placental weight, **(e)** fetal to placental ratio, **(f)** placental diameter, and **(g)** intra-placental uric acid levels in pooled placental samples; n = 8 mothers in allopurinol group, n = 12 in untreated group. **(h)** Fetal serum triglyceride levels; pooled serum from n = 4–6 mothers. Data are presented as mean and standard error of the mean. Identical letters indicate no statistical difference between groups, and different letters indicate significance (at least *P* < 0.05) as determined by 2-way ANOVA and Tukey post-hoc analysis.

To determine whether xanthine oxidase-induced uric acid was responsible for the adverse maternal-fetal outcomes of HFrD exposure, we treated the mice with the xanthine oxidase inhibitor allopurinol. Allopurinol can be supplied in the drinking water and can readily cross the placenta^31^. Not surprisingly, allopurinol treatment beginning three weeks before and throughout pregnancy did not reverse the pre-pregnancy glucose intolerance observed in HFrD-fed mice (Fig. 4a,b). Allopurinol treatment of HFrD-fed mothers improved fetal weights, but not significantly (*P* = 0.06, Fig. 4c). However, placental weight (Fig. 4d), placental diameter (Fig. 4f), fetal-to-placental weight ratio (Fig. 4e), and placental uric acid levels (Fig. 4g) were fully normalized in the allopurinol-treated HFrD-fed dams. Finally, the fetuses of allopurinol-treated HFrD-fed mice had serum triglyceride levels that were comparable to those of the controls (Fig. 4h). Together, these data suggest that excess uric acid in the placenta contributes to placental inefficiency and that maternal allopurinol treatment could ameliorate both placental effects and fetal hypertriglyceridemia resulting from maternal HFrD exposure.

### Placental uric acid is correlated with maternal serum fructose in human pregnancy

To explore the clinical relevance of our findings, we examined the relationship between maternal serum fructose and uric acid in term placentas from a small cohort of women (n = 18) undergoing non-laboring cesarean deliveries. Clinical characteristics of the women and babies are presented in Supplementary Table S2.

**Figure 5:**
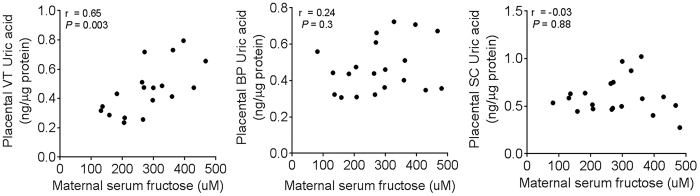
Human maternal serum fructose level correlates with placental villous tree uric acid level. VT = villous tree, BP = basal plate, SC = sub chorion, r = Pearson correlation coefficient; n = 18.

We measured uric acid levels in three different placental layers and found a significant correlation between maternal serum fructose and level of uric acid in the placental villous tree but not in the basal plate or sub chorion (Fig. 5). After accounting for a number of potential confounders (Supplementary Table S3), the association between maternal serum fructose and placental villous tree uric acid level remained stable and significant, suggesting that maternal serum fructose level is an independent predictor of placental villous tree uric acid levels. We then asked how well maternal serum fructose could predict placental villous tree uric acid levels as an outcome. Because of our small sample size, a multivariate analysis was not feasible; therefore, we ran a univariate linear regression analysis to examine the effect of maternal serum fructose in predicting the placental villous tree uric acid levels as an outcome. We used the log transformation of the outcome to meet the normality assumption of the linear regression model (Supplementary Table S4). The estimating strength of maternal serum fructose as a predictor was moderate with a R^2^ value of 0.44 and a 1.002 (ng/mg protein) increase in placental villous tree uric acid levels was found for every 1 mM increase in maternal serum fructose levels (*P* = 0.003). Because maternal serum fructose, but not serum uric acid, correlated with placental villous tree uric acid levels (Supplementary Table S4), our data suggest that increased circulating fructose in pregnant women drives placental uric acid production.

## Discussion

Increased fructose consumption has been linked to metabolic syndrome, but the effects of fructose consumption during pregnancy on maternal and offspring health are less well characterized. In this study, we used a murine model to test the effects of elevated fructose consumption on fetal-placental development. Maternal HFrD exposure led to increased uric acid in the placenta but not in the maternal serum, indicating that the placenta produced excess uric acid. Additionally, maternal HFrD exposure led to a pro-oxidative placental milieu, fetal growth restriction, and fetal hypertriglyceridemia. Many of these effects were normalized by treating the mothers with allopurinol, indicating that placental xanthine oxidase and uric acid are responsible for the fructose-induced placental and fetal impairments. Furthermore, our observation in a small cohort of women that maternal serum fructose correlated with levels of uric acid in placentas suggests that similar effects occur in humans.

Our findings in mice differ from those in rats on a high-fructose diet, which exhibit many features of metabolic syndrome such as hyperglycemia, hyperinsulinemia/insulin resistance, hypertension, hyperuricemia, and elevated plasma triglycerides^3^,^24^,^32^,^33^. Additionally, whereas we observed fetal growth restriction in HFrD-exposed mice, that effect was not observed in rats^21^,^22^. It is important to note that in our study, unlike several others in rodents, the mice on the HFrD consumed the same amount of food as those on the control chow diet and thus did not suffer from any nutritional deficits, especially in protein.

Consistent with findings in pregnant rats^22^, we found that pregnant mice on HFrD developed hepatic steatosis. This may explain the mild glucose intolerance we observed, as intrahepatic triglyceride accumulation can impair insulin-induced suppression of hepatic glucose production^34^. Interestingly, a recent study showed that women with non-alcoholic fatty liver disease are susceptible to adverse pregnancy outcomes such as low birth weight, gestational diabetes, and preeclampsia independent of body mass index or diabetes^35^. Thus, fructose-induced hepatic steatosis may have a negative impact on pregnancy in our mouse model.

Our findings may shed light on intrauterine growth restriction (IUGR) in humans. In mice, we found that maternal fructose consumption led to fetal growth restriction and placental inefficiency. Additionally, we found that the labyrinth zone (the vascular area of the placenta responsible for maternal-fetal nutrient, gas, and waste exchange) was significantly smaller in the HFrD-fed dams than in controls. Our data are consistent with human studies showing that IUGR is a consequence of utero-placental inefficiency^36^. Furthermore, we observed a correlation between maternal serum fructose and uric acid within the placental villous tree in humans, indicating that this zone, which is analogous to the murine labyrinth, is most susceptible to the negative effects of fructose. To our knowledge, this is the first study to measure placental uric acid levels in humans. Although it is well known that elevated serum uric acid is correlated with preeclampsia^37^ and IUGR^38^, serum uric acid has not proven to be an effective predictor of preeclampsia. In addition, the source of elevated maternal serum uric acid in women with preeclampsia is not well established and could be the fetus, placenta, or the maternal organs and vasculature^39^. None of our cohort of women had clinically diagnosed preeclampsia; however, our data suggests that maternal fructose could play a role in placental uric acid production, which could contribute to hyperuricemia observed in some preeclamptic patients. Consistent with this idea, a study of 32,933 nulliparous women showed that consumption of sugar-sweetened beverages was associated with an increased risk of preeclampsia^12^. Studies examining effects of the fructose-uric acid pathway in preeclamptic placentas are currently ongoing.

Although our data did not reveal the mechanism by which placental uric acid contributed to fetal growth restriction, one possibility is oxidative stress. Indeed, placental oxidative stress is associated with IUGR, pregnancies with growth-restricted fetuses have placental endothelial dysfunction^40^, and uric acid can promote endothelial dysfunction and inefficient placentation^4^,^41^. Additionally, xanthine oxidase activity is elevated in placentas from preeclamptic patients^42^, and xanthine oxidase can promote placental oxidative stress and apoptosis^43^, thereby contributing to placental dysfunction associated with preeclampsia^44^. In this study, treatment with allopurinol led to significantly lower placental uric acid levels and rescued the fructose-induced placental inefficiency without improving maternal glucose homeostasis, suggesting a direct effect of fructose-induced uric acid on placental function. However, xanthine oxidase can also induce oxidative stress through the generation of ROS and superoxide. Therefore, treatment with allopurinol may reduce overall levels of oxidative stress in a way that is not specific to increases in uric acid. Moreover, allopurinol is commonly used to treat hyperuricemia in humans, and one consequence of treatment is improved endothelial function^19^. Whether placental endothelial defects occur as a result of HFrD exposure in mice remains to be determined.

Several epidemiological studies^17^,^45^ have shown that IUGR or low birth weight increase the risk of developing metabolic syndrome, type 2 diabetes, and cardiovascular disease in adulthood. In this study, we did not follow the HFrD-exposed fetuses to adulthood. However, fetuses from HFrD-fed mice had elevated circulating glucose and lipids and were growth restricted, suggesting they were at increased risk of future cardio-metabolic diseases.

We acknowledge that uric acid homeostasis in rodents is unlike that of humans. However, humans have higher serum urate levels compared to rodents due to the genetic silencing of the hepatic enzyme uricase, which metabolizes uric acid into allantoin^27^. Thus, the negative effect of excess fructose in humans is likely to lead to an exacerbation of the mouse phenotype. In our human studies, we found that maternal serum fructose level was an independent predictor of placental villous tree uric acid and that the maternal serum uric acid level did not confound this relationship. This favors the notion that, as in mice, fructose may drive *de novo* production of uric acid in the human placenta. Although few data exist on placental fructose transport, we previously showed that the hexose transporters GLUT8^46^ and GLUT9^47^ can transport fructose and are expressed in human placentas^48^,^49^. This correlation between maternal serum fructose and placental uric acid should be confirmed in a larger cohort suitable for multivariate analyses, and the underlying mechanisms should be further investigated. Finally, our study is limited by the fact that term placentas were used to assess the relationship between serum fructose and placental uric acid. Since many of the placental disorders associated with oxidative stress (IUGR and preeclampsia) result from much earlier changes in placental development, the uric acid content at term is not necessarily reflective of the original insult. However, first and second trimester tissues are not readily available making it extremely difficult to preform such studies.

In conclusion, our work indicates a novel mechanism by which increased fructose consumption can negatively affect maternal-fetal outcomes. The impact of fructose consumption on human health has become increasingly important since the introduction of high fructose corn into the US food supply just before 1970. Now, high fructose corn syrup constitutes over 50% of the sweeteners used and Americans consume on average 26.8 pounds of HFCS per capita per year (http://www.ers.usda.gov/data-products/sugar-and-sweeteners-yearbook-tables.aspx). The work presented herein gives premise to the necessity of understanding the potentially negative effects of high fructose diets in humans, in particular during pregnancy.

## Methods

### Animal diet, breeding, and treatment

All procedures were performed in accordance with the approved guidelines by the Animal Studies Committee at Washington University School of Medicine. Six-week-old C57BL6/J female mice (Jackson Laboratory, Bar Harbor, ME) were fed a high-fructose diet (HFrD; Harlan Teklad, Madison, WI) or standard rodent chow ad libitum for six weeks. HFrD was similar to the control chow except that the complex carbohydrates (corn starch, maltodextrin, and 3% sucrose) were replaced with 60% fructose. The percent kilocalories obtained from carbohydrates (66.8% HFrD vs. 62.1% chow) and fat (12.9% HFrD vs. 13.2% chow) were similar between the two diets. Food intake was measured two or three times per week and averaged weekly and was similar between the two groups (Supplementary Fig. S1). Body weights were measured weekly throughout the duration of the study. After six weeks of feeding, both HFrD- and chow-fed mice were mated overnight to chow-fed C57BL6/J male mice. The male was removed once mating was confirmed via appearance of a vaginal plug the next morning (day post-coital [dpc] 0.5). Mice were maintained on their respective diets throughout pregnancy until they were sacrificed on dpc 18.5. For allopurinol treatment, mice were provided with drinking water containing 150 mg/L allopurinol three weeks before and during pregnancy. Mice were maintained on a 12-h light/dark cycle.

### Mouse glucose and insulin tolerance tests

Mice were fasted for six hours before glucose tolerance tests or four hours before insulin tolerance tests. Blood glucose was measured before mice were injected intraperitoneally with 2 mg of glucose per gram of body weight for glucose tolerance tests or 0.5 units of insulin per gram of body weight for insulin tolerance tests. The one-touch Bayer Contour T5 glucometer (Mishiwaka, IN) was used for all glucose measurements. The quantitative insulin-sensitivity check index (QUICKI) was used to determine insulin sensitivity in pregnant dams and fetuses where QUICKI = 1/(log(fasting insulin) + log(fasting glucose)) as described previously^50^.

### Mouse placental and fetal tissue collection

On dpc 18.5, blood pressure of the mothers was measured by using Columbus Instruments’ Non-Invasive Blood Pressure Monitor system (NIBP S/N 110707-1). Mothers were fasted for four hours before blood collection via the submandibular/facial vein. Mice were then euthanized in accordance with animal studies committee regulations. Individual fetal-placental units were dissected from the uterine horns, and fetal membranes were discarded before weighing and measuring. Placental weights, placental diameter, fetal weights, and crown-rump lengths were measured. Fetal sex was determined visually under a dissecting microscope by gonadal identification. Both male and female placentas were analyzed in all experiments and combined for data analysis, as no sex-based differences were observed. Placental tissue was rinsed in ice-cold PBS before snap freezing in liquid nitrogen or processing for procedures listed below. All samples were stored at −80 °C until use. Fetal trunk blood was collected and pooled from each litter. For all measurements, values from all fetuses or placentas in a litter were averaged and the reported n values reflect the number of mothers.

### Human tissue collection

All experimental protocols were performed in accordance with the guidelines and regulations at Washington University School of Medicine. Human tissue samples were collected by the Women and Infants’ Health Specimen Consortium and the collection protocol was approved by the Institutional Review Board at Washington University (201409097). A written informed consent was obtained from all subjects prior to tissue collection at term. Women participating in this study were chosen to represent a range of race, body mass index, and metabolic disease such that their defined characteristics followed a normal distribution pattern. All participants delivered full-term infants whose weights were normal for gestational age. Sera, placentas, and umbilical cord blood (mixed arterial and venous) were collected from pregnant women delivering at term at Barnes-Jewish Hospital. Included in this study were mothers who had non-laboring cesarean deliveries. Clinical characteristics were obtained from medical records. Three 5 mm^2^ samples were collected from each of three distinct placental zones – basal plate, villous tree, and sub-chorion – to ensure adequate coverage of the entire placenta. Non-fasting maternal serum was collected from participants at the time of delivery.

### Serum measurements

Serum levels of fructose (Intra-assay coefficient of variance, %CV = 3.4) and uric acid (%CV = 2.3) in humans were measured with the Fructose Assay Kit (#F8000-11, US Biologicals) and the Infinity Uric Acid Reagent (#TR24321, Thermo Scientific), respectively, according to manufacturers’ instructions. Serum triglyceride (%CV = 8.9) , cholesterol (%CV = 5.8), non-esterified free fatty acids (%CV = 1.9), and insulin (%CV = 4.6) were measured in mice fasted for four hours by using the infinity triglyceride & cholesterol reagent kit (Fisher Diagnostics), the NEFA-HR (2) assay kit (Wako Diagnostics), and the rat/mouse insulin ELISA kit (Crystal Chem, IL), respectively, according to the manufacturers’ instructions. The Washington University Mass Spectrometry Core Facility measured mouse serum uric acid levels as previously described^51^. Serum glucose (%CV = 2.1) and fructose (%CV = 1.3 levels) in mice were determined as previously described^46^,^52^.

### Quantitative real-time RT-PCR and Immunoblotting

Mouse placentas were individually snap-frozen and used for RNA extraction and subsequent PCR quantification or lysed for immunoblotting. RNA was isolated by using TRIzol reagent (Invitrogen). The Quantitect Qiagen reverse transcriptase kit (Qiagen, Valencia, CA) was used to synthesize cDNA. Quantitative PCR (7500/7500 fast real-time PCR system, software version 2.0.3, Applied Biosystems) was performed using the SYBR Green master mix (reagent from Applied Biosystems, Carlsbad, CA). The PCR reaction was carried out for 40 cycles of 95 °C for 20 s, 95 °C for 3 s, and 60 °C for 30 s. Quantification of gene expression was determined using the ΔΔCt approach normalizing to beta-actin. Primers used were: fructokinase - 5′TAC GAC ACG AAC CTG CCA GA3′ and 5′ CAC CTG TTC CGA TGC ATT CC3′, beta-actin – 5′ACC TTC TAC AAT GAG CTG CG3′ and 5′CTG GAT GGC TAC GTA CAT GG3′. Primer efficiency was determined by generating a standard curve from a cDNA pool of all our sample groups. The amplification efficiency was 96.8% for fructokinase and 94% for beta-actin primers. Primers were specific for the genes as the dissociation curves had single peaks with no shoulders and had melting temperatures above 80 °C indicating no primer-dimer formations. Beta-actin expression was independent of treatment.

For all immunoblot analysis, 20 μg of total protein lysate was loaded per lane. Immunoblots were incubated overnight in TBST/5% milk containing antibodies specific to the following proteins: Uricase (1:500; Santa Cruz Biotechnology, TX), Superoxide 2 (SOD2) (1:1000; Santa Cruz Biotechnology, TX), Glutathione peroxidase, catalase, beta-actin, 4HNE (all 1:1000; Abcam, MA), and cyclophilin B (1:5000; Abcam). Blots were washed and probed with goat secondary antibody conjugated to horseradish peroxidase (1:5000). Chemiluminescence was detected by using the Super Signal West Pico kit (Thermo Scientific).

### Histology and immunohistochemistry

After animal sacrifice, cross-sections of each placenta were cut and fixed overnight in 10% neutral buffered formalin (Fisher Scientific) or cryopreserved in optimal cutting temperature compound (Tissue-Tek). Formalin-fixed placentas were processed into paraffin blocks and cut into 5 μm sections for further histological analyses. The placental sections were stained with Hematoxylin and Eosin, and areas of placental zones were measured with ImageJ analysis software (version 1.47). The Vectastain Elite ABC kit (Vector labs, MI) was used for immunohistochemical detection. Primary antibodies recognized xanthine oxidase (1:100; Abcam, MA). Oil-red-o analysis of cryopreserved placentas was performed as previously described^46^ and quantified by ImageJ analysis software (version 1.47). An average of four or five placentas per mother and at least five mothers in each group were used for all the above analyses.

### Placental metabolite quantification

To measure mouse and human placental uric acid concentration, placental tissue was homogenized in PBS and centrifuged at 4 °C. The internal standard (1 ng/μl of 1-methyl uric acid) and 70% methanol were immediately added to the supernatant, which was then dried under a stream of nitrogen and re-dissolved in 500 μl of water. Analysis was performed as previously described^51^ at the Washington University Mass Spectrometry Core Facility. ATP levels in mouse placentas were measured as previously described^52^. To determine XO and AMPD activity in mouse placentas, each fresh placenta was homogenized in ice-cold 100 mM Tris HCl, 1 mM EDTA, pH7.5, and centrifuged at 9,300xg for 10 minutes. The supernatant was used to quantify XO activity according to the manufacturer’s instructions (Cayman Chemicals, MI). Before measuring AMPD activity in placental samples, two reagent mixtures were prepared and pre-treated. First, the 2X AMPD reagent (60 mM Imidazole base, 25% glycerol, 0.1% fraction V & protease-free BSA, 2 mM DTT, 2 mM ATP, 10 mM 5′-AMP, and 200 mM KCl) was pre-treated with 25 mM acetic anhydride for 10 minutes at room temperature. Second, 2X NH4 reagent (100 mM Trizma base, 0.04% fraction V & protease-free BSA, 200 μM ADP, 2 mM α-ketoglutarate) was pre-treated with 12.5 mM acetic anhydride at room temperature for 10 minutes. After pre-treatment, 400 μM NADH and 160 μg/ml GDH in glycerol was added to the mix. To measure AMPD activity, the placental supernatant was diluted 1:400 in 20 mM Imidazole HCl, pH7.0, and 0.05% fraction V & protease-free BSA. A 20 μl sample was added to 20 μl of pre-treated 2X AMPD reagent and incubated at 37 °C for 60 minutes. The reaction was stopped by adding 10 μl of 0.15 N HCl and incubating at 60 °C for 20 minutes. Next, 50 μl of pre-treated 2X NH4 reagent was added, and the mixture was incubated at room temperature for 25 minutes. The reaction was terminated by adding 10 μl of 0.6 N HCl and incubating for 10 minutes at room temperature. A 10 μl aliquot was added to 1 ml of 6 N NaOH and 10 mM Imidazole base, mixed immediately, and incubated at 60 °C for 20 minutes for strong base fluorescence enhancement. Fluorescence was detected with an A-1 FOCI fluorometer (Valhalla, NY) at 340 nm with internal standards generated from (NH4)2SO4. All of the above reagents were purchased from Sigma-Aldrich (St. Louis, MO).

### Statistics

Data are expressed as mean ± SEM. GraphPad PRISM version 6.0 (La Jolla, CA) was used to perform either Student’s t-tests assuming two-tailed distribution and unequal variances for comparison between two groups or two-way ANOVA analyses for multiple comparisons. Correlations and univariate linear regression analyses were performed by the Institute of Clinical and Translational Sciences Biostatistics division at Washington University in St. Louis using SAS 9.3. For the univariate linear regression analysis, the log transformation of placental villous tree uric acid levels was used because the data were not normally distributed. For all data, *P* < 0.05 was considered statistically significant.

## Additional Information

**How to cite this article**: Asghar, Z. A. *et al.* Maternal fructose drives placental uric acid production leading to adverse fetal outcomes. *Sci. Rep.* **6**, 25091; doi: 10.1038/srep25091 (2016).

## Supplementary Material

Supplementary Information

## Figures and Tables

**Table 1:** Maternal and fetal fasting serum measurements on day post-coital 18.5.

Parameter	Maternal Serum		Fetal Serum	
	Control	HFrD	Control	HFrD
Fructose (μM)	63.3 ± 5.1	885.3 ± 274.5**	36.8 ± 4.0	119.2 ± 27.6**
Glucose (mM)	5.94 ± 0.28	8.52 ± 0.77**	0.89 ± 0.13	1.6 ± 0.17**
Insulin (ng/mL)	0.96 ± 0.16	0.87 ± 0.14	0.29 ± 0.04	0.36 ± 0.04
QUICKI	0.29 ± 0.01	0.28 ± 0.01	0.44 ± 0.02	0.45 ± 0.05
Uric acid (μg/mL)	7.89 ± 1.65	10.95 ± 0.98	1.20 ± 0.31	1.24 ± 0.18
Triglyceride (mM)	0.54 ± 0.03	0.38 ± 0.03**	0.32 ± 0.02	0.42 ± 0.03**
Cholesterol (mM)	1.39 ± 0.1	0.92 ± 0.07***	1.30 ± 0.12	1.14 ± 0.09
NEFA (mM)	1.09 ± 0.14	0.89 ± 0.12	0.29 ± 0.02	0.29 ± 0.01

QUICKI: Quantitative insulin sensitivity check index; NEFA: Non-esterified fatty acid; Data are expressed as mean ± SEM of n = 6–10 mothers per maternal group. For fetal serum measurements, the litter sera were pooled per mother; **P < 0.01, ***P < 0.001 by Student’s *t-*test.
