# Supplementary material for: Maternal fructose drives placental uric acid production leading to adverse fetal outcomes
Source: Sci Rep. 2016 Apr 29;6:25091. doi: 10.1038/srep25091 (PMC4850405; doi:10.1038/srep25091)
Supplement: Supplementary Information [file srep25091-s1.pdf]

Supplementary information for:

Maternal fructose drives placental uric acid production leading to adverse fetal outcomes

Zeenat A. Asghar[a], Alysha Thompson[a], Maggie Chi[a], Andrew Cusumano[a], Suzanne Scheaffer[a], Noor Al-Hammadi[b], Jessica L. Saben[a] and Kelle H.Moley[a]

[a] Department of Obstetrics & Gynecology, [b] Department of Biostatistics, Washington University in St. Louis School of Medicine, St. Louis, Missouri, USA

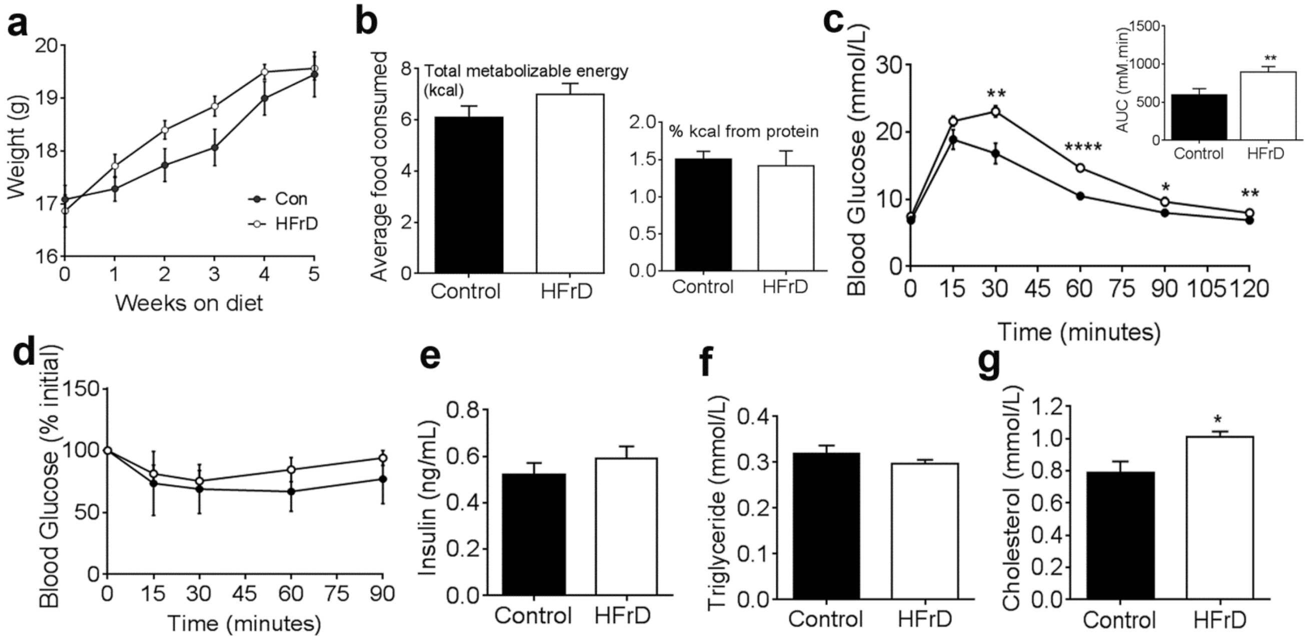

Supplementary Figure S1. Six weeks of high-fructose diet leads to impaired glucose homeostasis but not insulin resistance in mice. Mice were fed standard chow diet (control, filled circles and bars) or 60% fructose diet (HFrD, open circles and bars) for six weeks. **(a)** Body weights of HFrD- and control chow-fed mice. **(b)** Average food consumption by chow-fed (control) and high-fructose-fed (HFrD) mice was similar as total kilocalories (kcal) and percent of kcal from protein received per mouse per day; n= 10 mice in each group. **(c)** Blood glucose levels over time following an intraperitoneal injection of 2 mg/g glucose; n= 12 in each group. Inset= area under the curve. **(d)** Blood glucose levels following an intraperitoneal injection of 0.5 U insulin; n=6 in each group. **(e-g)** Fasting serum insulin (**e**), triglyceride (**f**), and cholesterol (**g**) levels; n=10 in each group. Data are presented as mean and standard error of the mean. *$P<0.05$, ** $P<0.01$, **** $P<0.0001$ by Student's *t*-test.

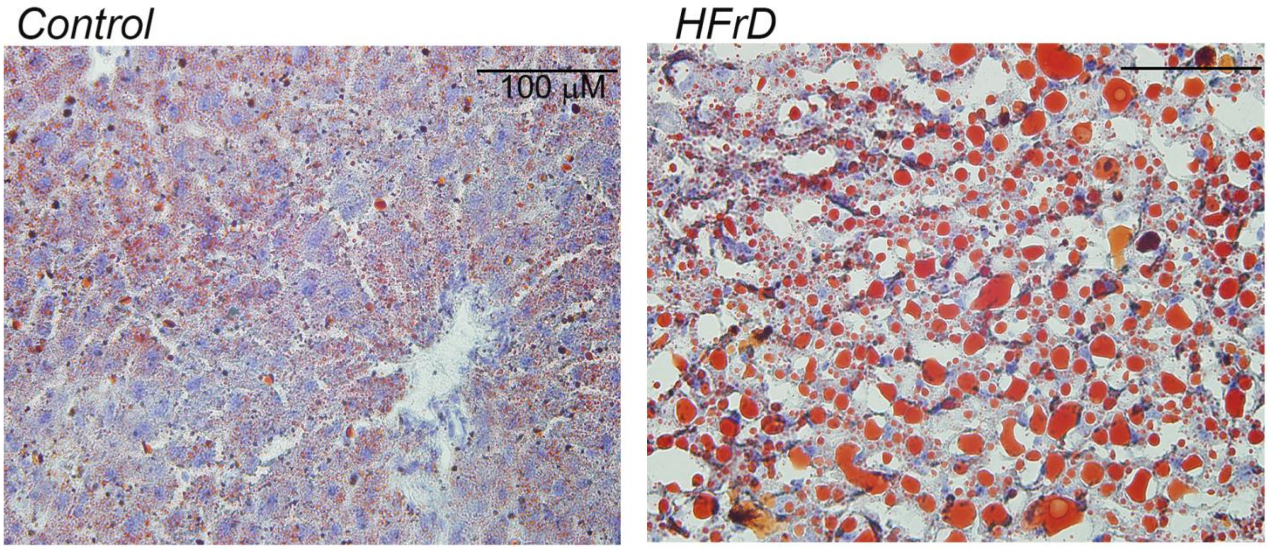

Supplementary Figure S2. High-fructose diet consumption induces hepatic steatosis. Representative image of Oil red-O staining of liver from chow-fed (control) and high-fructose-fed (HFrD) pregnant mice collected on day post-coital 18.5.

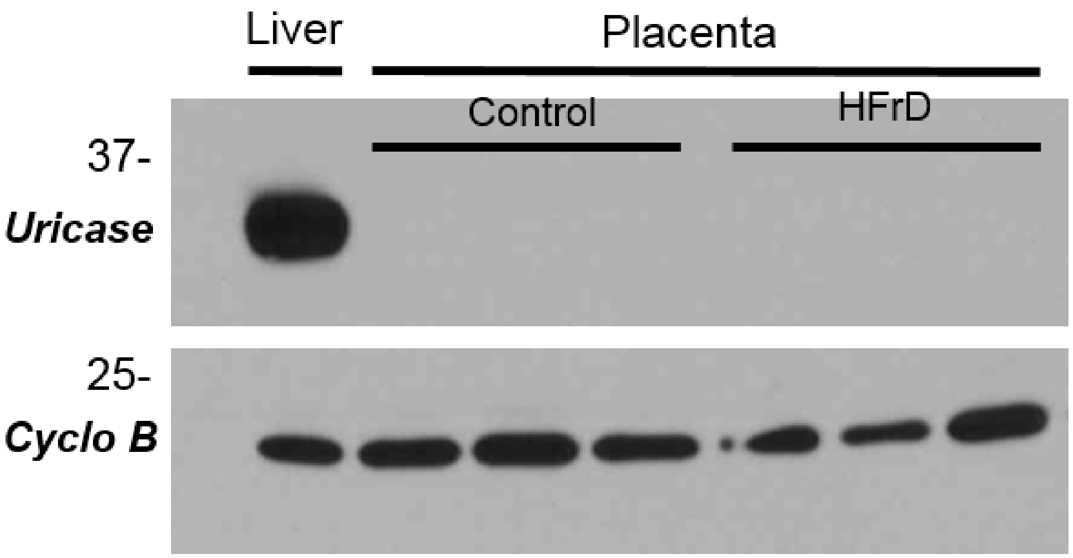

Supplementary Figure S3. The hepatic enzyme uricase is not expressed in mouse placenta. Western blot showing expression of uricase in liver (34 kDa) as well as placentas from chow-fed (control) and high-fructose-fed (HFrD) pregnant mice collected on day post-coital 18.5; Cylo B, cyclophilin B (22 kDa).

Supplementary Table S1 - Blood pressure measurement by tail-cuff plethysmography in pregnant mice on day post-coital 18.5.

	Control	High fructose
**Systolic**	108 ± 6	107 ± 2
**Diastolic**	69 ± 5	76 ± 3
**Mean**	83 ± 5	85 ± 2

Data are expressed as mean and standard error of the mean; n= 6-8 mothers in each group

Supplementary Table S2 - Variables in the analysis.

**Variable**	**Mean[1]**	**Standard Deviation**	**Median**	**IQR**
Age (years)	29.1	6.3	30.5	7
Body mass index (kg/m^2)	33.5	8.8	32.4	7.3
Gestational age (weeks)	38.8	1.3	39	2
Maternal serum fructose (µmol/L)	279.1	98.7	270	154
Maternal serum uric acid (µmol/L)	231.1	63.8	243.6	74.2
Cord uric acid (µmol/L)	256.1	36.6	259.5	33.2
Cord fructose (µmol/L)	465.9	147.8	432	136
Birth weight (grams)	3403	419.3	3352.5	545
Birth length (cm)	50.2	2.2	50.8	3.5
APGAR 1 min	7.5	1.7	8	1
APGAR 5 min	8.9	0.2	9	0
Placental uric acid (ng/µg protein)– villous tree	0.5	0.2	0.5	0.2
Placental uric acid (ng/µg protein)– basal plate	0.5	0.1	0.4	0.3
Placental uric acid (ng/µg protein)- sub chorion	0.6	0.2	0.6	0.2
Diabetes?				
No	14 (77.8)			
Type 2 diabetes mellitus	3 (16.7)			
Gestational diabetes	1 (5.6)			
Hypertension?				
No	13 (72.2)			
Chronic hypertension	3 (16.7)			
Gestational hypertension	2 (11.1)			

IQR = interquartile range; n= 18 subjects

[1]Diabetes and Hypertension data are presented as n (%)

Supplementary Table S3 - Correlation between the main independent predictor (maternal serum fructose) and the outcome (placental villous tree uric acid) after adjustment for confounders.

**Confounder**	**Correlation**	***P*-value**
Age	0.629	0.0068
Body mass index	0.671	0.0032
Diabetes Mellitus	0.614	0.0087
Hypertension	0.657	0.0042
Maternal serum uric acid (µmol/L)	0.665	0.0036
Gestational age (weeks)	0.663	0.0037

Supplementary Table S4 - Univariate linear regression analysis of maternal serum fructose and confounders with the outcome (placental villous tree uric acid)

	Univariate Analysis			
**Predictor**	**Estimate**	**Standard Error**	***P*-value**	**R-Square**
Maternal serum fructose (μmol/L)	0.0025	0.0007	0.003	0.444
Maternal uric acid (μmol/L)	0.0018	0.0014	0.213	0.095
Cord uric acid (μmol/L)	0.0009	0.0025	0.740	0.007
Age	-0.0181	0.0140	0.216	0.094
Body mass index	-0.0069	0.0104	0.519	0.026
Has Diabetes Mellitus	-0.2489	0.2078	0.249	0.082
Has Hypertension	-0.0764	0.2005	0.708	0.009
